# Persistent resistance to HIV-1 infection in CD4 T cells from exposed uninfected Vietnamese individuals is mediated by entry and post-entry blocks

**DOI:** 10.1186/1742-4690-3-81

**Published:** 2006-11-08

**Authors:** Asier Sáez-Cirión, Pierre Versmisse, Lien X Truong, Lisa A Chakrabarti, Wassila Carpentier, Françoise Barré-Sinoussi, Daniel Scott-Algara, Gianfranco Pancino

**Affiliations:** 1 Unité de Régulation des Infections Rétrovirales, Institut Pasteur, Paris, France; 2 Retrovirology and Viral Hepatitis Laboratory, Institut Pasteur, Ho Chi Minh City, Vietnam; 3 Laboratoire de Pathogénie Virale Moléculaire, Institut Pasteur, Paris, France; 4 Laboratoire d'Immunologie Cellulaire, UR INSERM 543, Faculté de Médecine Pitié-Salpétrière, Paris, France; 5 Unité d'Immunogénétique Cellulaire, Institut Pasteur, Paris, France

## Abstract

**Background:**

We have previously reported that CD4 T cells from some exposed uninfected (EU) Vietnamese intravenous drug users are relatively resistant to HIV infection in vitro. Here, we further characterized the restriction of viral replication in CD4 T cells from five EUs and assessed its persistence in serial samples.

**Results:**

CD4 T cells and/or PBMC sampled during a period of between 2 and 6 years were challenged with replication-competent HIV-1 and other retroviral particles pseudotyped with envelope proteins of various tropisms. CCR5 expression and function in resistant CD4 T cells was evaluated. The step at which HIV-1 replication is restricted was investigated by real-time PCR quantification of HIV-1 reverse transcripts.

We identified three patterns of durable HIV-1 restriction in EU CD4 T cells. CD4 T cells from four of the five EU subjects were resistant to HIV-1 R5 infection. In two cases this resistance was associated with low CCR5 surface expression, which was itself associated with heterozygous CCR5 mutations. In the other two cases, CD4 T cells were resistant to HIV-1 R5 infection despite normal CCR5 expression and signaling function, and normal β-chemokine secretion upon CD4 T cell activation. Instead, restriction appeared to be due to enhanced CD4 T cell sensitivity to β-chemokines in these two subjects. In the fifth EU subject the restriction involved post-entry steps of viral replication and affected not only HIV-1 but also other lentiviruses. The restriction was not overcome by a high viral inoculum, suggesting that it was not mediated by a saturable inhibitory factor.

**Conclusion:**

Various constitutive mechanisms of CD4 T cell resistance to HIV-1 infection, affecting entry or post-entry steps of viral replication, are associated with resistance to HIV-1 in subjects who remain uninfected despite long-term high-risk behavior.

## Background

Cellular susceptibility to human immunodeficiency virus (HIV) infection in vitro varies widely among individuals [1,2]. Both host genetic and acquired mechanisms regulate HIV-1 replication. HIV requires numerous host cell factors for efficient replication [3]. The recent discovery of several molecules endowed with antiretroviral activity in mice and primates underlines the contribution of innate intracellular resistance to infection by HIV and other retroviruses [4]. Some of these molecules, such as the cytidine deaminase APOBEC3G, have been implicated in the restriction of HIV-1 replication in resting human T cells [5,6]. Resistance to HIV-1 infection in vivo has not so far been linked to the expression or genetic polymorphism of these restriction factors [7,8], but the efficiency of viral replication is likely to be determined in large part by the balance between required factors and restrictive factors.

Some individuals who are highly exposed to HIV-1 and yet remain uninfected (exposed uninfected individuals, EU) are likely to be naturally resistant to infection. Relative resistance of CD4 T cells and/or macrophages to HIV-1 infection has been reported in selected EUs [9-11]. This resistance was usually restricted to HIV-1 isolates using the CCR5 chemokine receptor (R5 isolates) to enter target cells [12-14]. Invalidating mutations in the CCR5 gene confer resistance to HIV-1 R5 infection in vitro [15,16], and the CCR5Δ32 homozygous genotype is associated with protection against HIV-1 acquisition in Caucasians [17]. Reduced in vitro susceptibility to HIV-1 R5 of EU CD4 T cells bearing wild-type CCR5 has been linked to low CCR5 expression on the target cell surface and/or to increased secretion of natural CCR5 ligands – the β-chemokines RANTES/CCL5, MIP-1α/CCL3 and MIP-1β/CCL4 [12] – by CD4 or CD8 T lymphocytes [18-20]. Infection of CD4 T cells may also be inhibited by unidentified soluble antiviral factors secreted by CD8 T lymphocytes [21]. Nevertheless, CD8 T cell associated resistance to HIV-1 infection was reported to wane in EUs who reduced their high-risk behavior, suggesting that reduced exposure led to decreased CD8 T cell antiviral immunity [22,23].

We have previously shown that some Vietnamese intravenous drug users who remained uninfected by HIV despite more than 15 years of drug use (resulting in a high prevalence of other blood-borne viral infections) have low CD4 T cell permissiveness to HIV infection in vitro [11]. In order to identify the mechanisms of CD4 T cell resistance in this population, we investigated the characteristics of HIV-1 restriction in five Vietnamese EUs who were monitored for between 2 and 6 years. We identified three different patterns of restriction, affecting viral entry or post-entry steps. We also found that CD4 T cell resistance to HIV was stable over time.

## Results

### CD4 T cell resistance to single-round HIV-1 infection in Vietnamese EUs

In a previous study of Vietnamese IDU EUs we identified some individuals whose CD4 T lymphocytes showed reduced susceptibility to in vitro infection by replicative strains of HIV-1 [11]. HIV-1 replication in CD4 T cells from three EUs (W276, W278 and B195) was far less efficient than in CD4 T cells from healthy controls. Analysis of the *CCR5 *gene in the same population revealed heterozygous mutations in some subjects [24,25]. In two cases (B184 and W336) the mutations in CCR5 were associated with reduced co-receptor function in transfected cell lines [24,26], but the effect of these mutations in heterozygous primary cells was not assessed.

**Figure 1:**
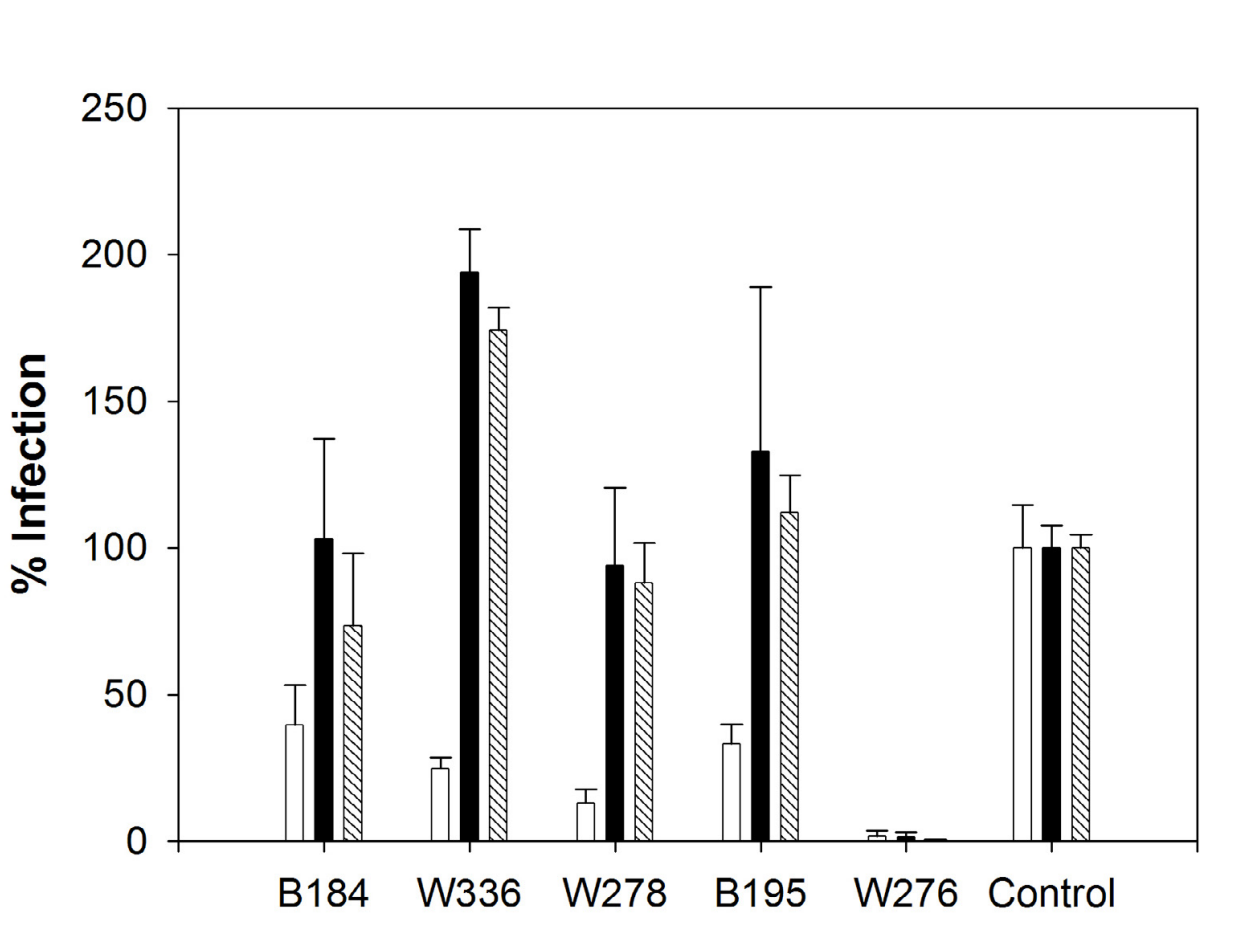
**Reduced susceptibility of EU CD4 T cells to infection by pseudotyped HIV-1**. CD4 T cells were challenged with HIV-1 particles pseudotyped with R5 (BaL) (white bars), X4 (HxB2) (black bars) or pantropic (VSV-G) (patterned bars) envelope glycoproteins. Results (mean of three experiments) are expressed as relative luciferase activity in cell lysates three days after infection. Luciferase activity in cell lysates from a representative control was attributed a value of 100%. Error bars representing standard deviation are shown in each case.

CD4 T cells from the five EUs studied here (W276, W278, B195, B184 and W336; Table 1) had reduced susceptibility to infectious strains of HIV-1 (data not shown and [11]; see Materials and Methods for corresponding subjects designations). We used single-round infection with envelope-pseudotyped HIV-1 NL4.3Δ env particles bearing the luciferase reporter gene to investigate whether the reduced HIV-1 susceptibility of the EUs' CD4 T cells was detectable during the first cycle of viral replication (fig. 1). In four out of five cases (W278, B195, B184, W336), CD4 T cells were less susceptible to infection by a CCR5-tropic (R5) HIV-1 pseudotype (HIV-BaL), while they were permissive to infection by a CXCR4-tropic (X4) HIV-1 pseudotype (HIV-HxB2) and to HIV-1 particles pseudotyped with the G protein from vesicular stomatitis virus (HIV-VSVG), which has a ubiquitous (pantropic) receptor and uses an endocytic entry pathway [27] (fig. 1). These results showed that the CD4 T cells of the EUs with heterozygous mutations in CCR5 (B184 and W336) were less susceptible to infection by HIV-1 R5 pseudotype and confirmed our previous observation that HIV-1 restriction in subjects W278 and B195 is specific to R5 viruses [11]. As shown, single-round infection of CD4 T cell from these four EUs was partially reduced. Interestingly, when replication-competent HIV-1 strains were used, inhibition of infection was much stronger (data not shown and [11]), suggesting that the restrictions observed during the first round of HIV-1 replication are amplified during subsequent cycles.

**Table 1:** Characteristics of the study subjects.

EU code	Year of birth	Sex	IDU started	Risk ended	Serology				Enrollment	Follow-up ended
					HBc	HBs	HCV	HTLV	Year	Year

W276	1960	M	1979	2003	+	-	+	+	1996	2004
W278	1955	M	1978	2000	-	-	+	+	1996	2004
W336	1958	M	1975	2001	+	-	+	+	1996	2004
B184	1951	M	1972	2003	+	-	+	+	1998	2004
B195	1949	F	1972	Unknown	+	-	+	-	1998	1999

HBc: anti-hepatitis B virus antibodies; HBs: hepatitis B antigen; HCV: hepatitis C virus; HTLV: human T cell leukaemia/lymphoma virus (I and/or II)

Remarkably, CD4 T cells from subject W276 were resistant to both R5 and X4 HIV-1 pseudotypes and also to the HIV-VSVG pseudotype. These results indicated that the restriction was independent not only of HIV coreceptors, as previously shown [11], but also of the entry pathway used by the virus (fig. 1).

### CCR5 expression and function in HIV-1 R5 restricted cells

As already mentioned, CCR5 heterozygous mutations had been detected in subjects B184 and W336 (G106R and C178R respectively) [24,25]. These (or equivalent) mutations, when present in the homozygous state in transfected cell lines, affect the receptor conformation and both CCR5 membrane trafficking and function [24,26]. However, CCR5 surface expression by these two EUs' primary CD4 T cells has not been evaluated before.

**Figure 2:**
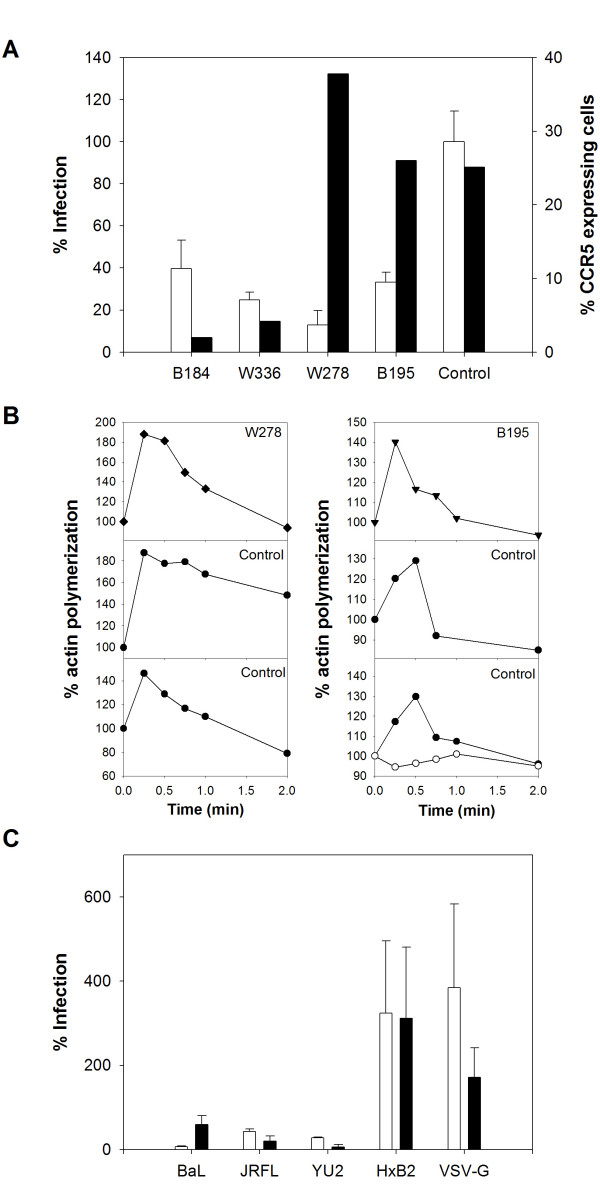
**R5 tropic HIV-1 restriction in CD4 T cells from four EUs**. **A. **Relative infection by the HIV-BaL pseudotype (white bars; n = 3, mean ± SD) of CD4 T cells from the EUs B184, W336, B195 and W278, and percentage of cells with detectable surface expression of the CCR5 co-receptor (black bars, one experiment shown, representative of two different experiments). **B. **CCR5-mediated actin polymerisation in CD4 T cells from W278 (◆) B195 (▼) (top left and right panels respectively) and four different CCR5-wt controls. Cells from a control donor (bottom right panel) were also treated with TAK-779 (2 μM) for 60 minutes before RANTES stimulation (open circles). Results show the kinetics of actin polymerization triggered by RANTES stimulation, as measured by the incorporation of the FITC-phalloidin probe. The percentage of actin polymerization is expressed as follows: [(MFI after ligand addition)/(MFI before ligand addition)] × 100. 100% corresponds to the baseline level of unstimulated cells. **C. **Relative infection of CD4 T cells from subjects W278 (white bars) and B195 (black bars) by R5 (HIV-BaL; HIV-JRFL; HIV-YU2), X4 (HIV-HxB2) and pantropic (HIV-VSVG) pseudotypes (n = 3, mean ± SD). The luciferase activity in cell lysates from one representative control was attributed a value of 100%.

Flow cytometry of R5-restricted CD4 T cells revealed that the percentage of CD4 T cells expressing detectable surface CCR5 was far lower in the two EUs carrying heterozygous CCR5 mutations (B184 and W336) than in controls expressing the wild-type (wt) CCR5 molecule (fig. 2A). In contrast, no such difference was found, in either the percentage (fig. 2A) or the mean fluorescence intensity (MFI), in the other two EUs (W278 and B195) who both had wt CCR5 (the CCR5 MFI was 1.38 and 1.21 in subjects W278 and B195, respectively, and 1.39 ± 0.24, mean ± SD, in five CCR5-wt controls).

Therefore, the low surface expression of CCR5 on CD4 T cells from EUs B184 and W336 is likely linked to CCR5 mutations and appears to affect R5 virus entry into target cells. However, HIV R5 replication in CD4 T cells from subjects W278 and B195 was restricted despite normal CCR5 surface expression (fig. 2A). We therefore examined whether CCR5 function was impaired in the CD4 T cells of these two subjects, affecting signaling events potentially involved in HIV-1 replication [28,29].

As actin cytoskeleton reorganization is a major characteristic of chemokine responses, we analyzed CCR5-mediated actin polymerization in CD4 T cells from subjects W278 and B195. RANTES stimulation of CD4 T cells induced a rapid increase in the F-actin content of cells from the two EUs and from four CCR5-wt controls (fig. 2B). The peak responses occurred 15–30 s after stimulation, in keeping with a fully functional chemoreceptor [30]. Cell pretreatment with the CCR5 inhibitor TAK-779 [31] abrogated actin polymerization.

The restriction in CD4 T cells from subjects W278 and B195 affected HIV-1 viruses pseudotyped with different R5 tropic envelopes (JRFL [32] and YU2 [33]) (fig. 2C). These results confirmed that the restriction in W278 and B195 CD4 T cells is specific for the CCR5 entry pathway and indicated that it is independent of CCR5 expression and function.

### Abrogation of viral restriction in cells from subjects W278 and B195 with anti-β-chemokines

**Figure 3:**
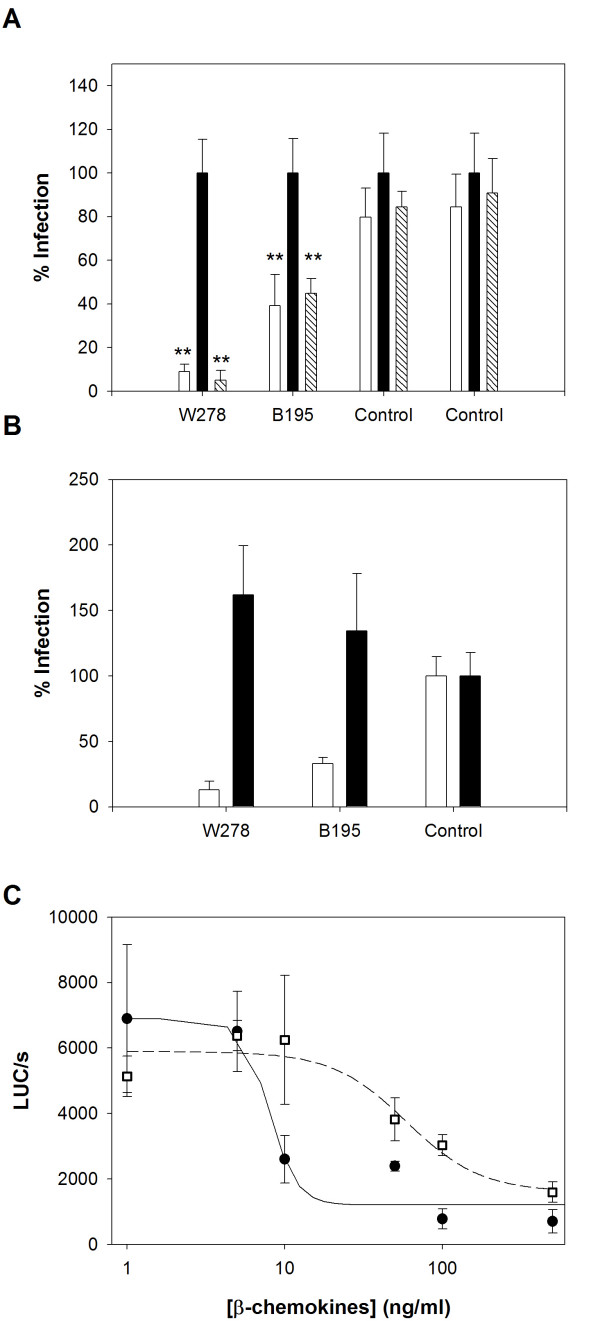
**Role of β-chemokines in HIV-1 restriction in CD4 T cells from subjects W278 and B195**. **A. **CD4 T cells from subjects W278 and B195 were challenged with the HIV-BaL pseudotype in the absence (white bars) or presence of a combination of neutralizing anti-RANTES (5 μg/ml), anti-MIP1α (15 μg/ml) and anti-MIP1β (25 μg/ml) (R&D systems, France) mAbs (black bars) or with an isotype control antibody (45 μg/ml) (patterned bars). The antibodies were added 30 minutes before challenge and maintained throughout the time course of infection. Results are expressed as relative luciferase activity, compared to the maximal activity found in the presence of the neutralizing anti-β-chemokines in each case, and are the mean of three independent infections ± standard deviation. ** Significant difference (P < 0.001 and P = 0.008 for W278 and B195, respectively, independent sample t-test). **B. **Challenge with the HIV-BaL pseudotype (n = 3, mean ± SD) of CD4 T cells from EUs W278 and B195, stimulated with PHA three days before (white bars) or two hours after (black bars) challenge. Luciferase activity in cell lysates from a representative control challenged in the same conditions was attributed a value of 100%. **C. **Sensitivity of CD4 T cells to recombinant chemokines. Non mitogen-stimulated CD4 T cells from EU B195 (filled circles) and one control (open squares) were exposed to various concentrations of a mixture of the recombinant β-chemokines RANTES, MIP-1α and MIP-1β (R&D systems, France) for 30 minutes prior to and during infection. The mixtures contained the three chemokines at concentrations ranging from 500 ng to 2 ng each. Results (n = 3, mean ± SD) are expressed as luciferase activity per second in cell lysates. Points were fitted to a four-parameter logistic curve (r^2 ^were 0.845 and 0.826 for B195 and control, respectively). Statistical analysis and curve-fitting were performed with Sigmaplot software (Systat Software, Inc, CA, USA).

R5 virus entry into CD4 T cells can be blocked by endogenously produced β-chemokines [20]. We therefore investigated whether the restriction of HIV-1 R5 replication in CD4 T cells from subjects W278 and B195 could be overcome by neutralizing monoclonal antibodies (MAbs) to RANTES, MIP1α and MIP1β. The addition of anti-β-chemokine mAbs, but not of irrelevant IgGs, strongly enhanced the infection of both W278 and B195 cells by the HIV-1 pseudotype, while no significant enhancement was observed in CCR5-wt control cells with similar CCR5 surface expression (fig. 3A). These results suggest that endogenously produced β-chemokines may be responsible for the inhibition of HIV-1 infection in CD4 T cells from subjects W278 and B195.

When we challenged CD4 T cells with BaL pseudotyped HIV in a setting of weak β-chemokine production (before PHA and IL2 activation; <3 ng) we found that infection was as efficient in the two EUs as in controls (fig. 3B). This was consistent with the hypothesis that the inhibition of HIV-1 infection in PHA-activated CD4 T cells from subjects W278 and B195 involved chemokines produced upon cell activation. However, quantification of β-chemokines produced by PHA-activated CD4 T cells at the time of HIV-1 infection (three days after PHA stimulation) showed no significant increase in β-chemokine secretion in W278 and B195 cell cultures compared to controls (Table 2).

**Table 2:** β-chemokines produced by mitogen-activated CD4 T cells.

EU code	RANTES (ng/ml)	MIP1α (ng/ml)	MIP1β (ng/ml)
W278	33.7	26.4	39.1
B195	19.7	56.3	66.8

Controls^a^	14.1 (11.7–34.3)	30.6 (26.8–49.8)	58.3 (45.5–67.3)

^a ^median and range for the 5 controls used in the experiments shown in figures 2 and 3.

To investigate the possibility of enhanced sensitivity to β-chemokines, we infected non-stimulated CD4 T cells from subject B195 (not enough cells from subject W278 were available) in the presence of a cocktail of recombinant RANTES, MIP1α and MIP1β added at increasing concentrations. In the presence of low levels (≤ 5 ng) of recombinant β-chemokines, HIV-1 replication was comparable in B195 and CCR5-wt control CD4 T cells (Fig. 3C), as already observed in the absence of added β-chemokines (Fig. 3B). In contrast, when the β-chemokine levels were increased, the efficiency of infection fell sharply in B195 CD4 T cells and far less markedly in control CD4 T cells (fig. 3C) (ID_50 _values were 8.12 ± 1.58 ng/ml and 59.34 ± 16.87 ng/ml for B195 and control respectively). CD4 T cell CCR5 surface expression was similar in the two individuals (data not shown). These results indicated that HIV infection of CD4 T cells from EU subject B195 was unusually susceptible to inhibition by β-chemokines.

### Pantropic restriction of HIV replication in subject W276 affects several lentiviruses

The blockade of in vitro HIV infection in CD4 T cells from subject W276 was independent of viral tropism and of the entry pathway (fusion or endocytosis) (fig. 1). In a fluorimetric fusion assay with cells expressing HIV-1 envelope proteins [34], W276 CD4 T cells showed normal membrane fusion (not shown), further supporting post-entry restriction of viral replication in these cells.

**Figure 4:**
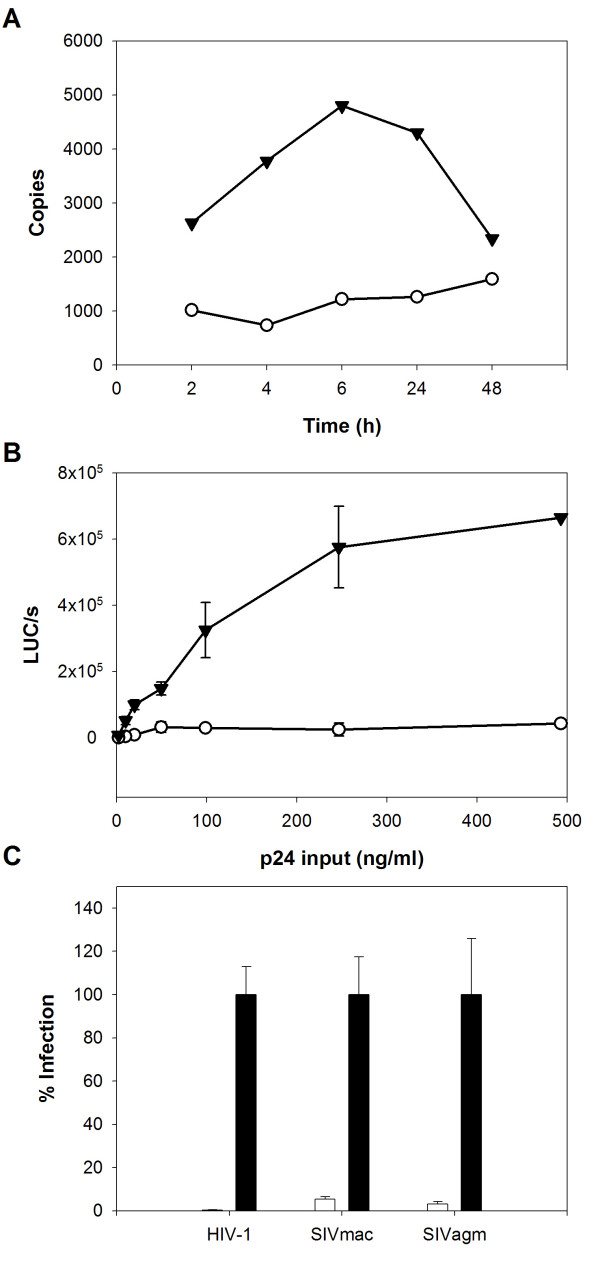
**Pantropic restriction in CD4 T cells from subject W276 affects the replication of several retroviruses**. **A. **Early reverse transcripts (RU5) analyzed by real-time PCR at various times after HIV-VSVG pseudotype challenge of CD4 T cells from subject W276 (open circles) and a representative control (filled triangles). **B. **Infection of CD4 T cells from subject W276 (open circles) and a control (filled triangles) with increasing amounts of HIV-VSVG pseudotype. Results (mean of three independent infections) are expressed as luciferase activity per second in cell lysates three days post-challenge. The control is representative of cells from three different controls. Error bars represent the standard deviation. **C. **Relative infection of CD4 T cells from subject W276 (white bars) by HIV-1, SIVmac and SIVagm particles pseudotyped with the VSVG fusion protein (n = 3, mean ± SD). Luciferase activity in cell lysates from a representative control (black bars) was attributed a value of 100%.

Previous qualitative PCR analysis of viral replication in CD4 T cells from subject W276 suggested that the restriction step occurred before integration [11], but early times post-infection were not analyzed. We therefore used single-round infection and real-time PCR to determine the precise stage at which the restriction occurred. Early reverse transcription products (R-U5) were far lower in W276 cells than in control cells, from the very first hours after infection (fig. 4A), and they increased very little during the time course of infection. Levels of PCR products corresponding to subsequent replication steps were also decreased (not shown). These results suggest that early post-entry steps of viral replication, most likely involving reverse transcription, are impaired in W276 CD4 T cells. Note that W276 CD4 T cells were readily activated by PHA (>95% of cells were CD25+ at the time of challenge) thus discarding that the restriction of HIV-1 infection was caused by a defect of the response to PHA-stimulation. In addition, HIV-1 restriction in W276 CD4 T cells was not overcome by increasing the size of the inoculum of VSV-G pseudotyped HIV-1 (fig. 4B), arguing against a role of a saturable restriction factor.

To determine whether the restriction of viral replication in CD4 T cells from subject W276 was specific to HIV-1 or also affected other lentiviruses, we challenged the cells with SIVagm and SIVmac luciferase reporter viruses (fig. 4C) pseudotyped with VSV-G. Replication of both viruses was strongly inhibited in W276 CD4 T cells.

### Persistence of HIV-1 restriction in primary cells from Vietnamese EUs

To determine whether resistance to infection was transient or persistent, we tested primary CD4 T cells or PBMC obtained from the five EUs at various times during 2–6 years of follow-up. The samples included cells obtained from four of the subjects (W276, W278, W336 and B184) after alleged interruption of IV drug use (Table 3). In infectivity assays with PHA-activated cells, HIV-1 replication was always inhibited in the EU cells compared to control cells. Moreover, the same pattern of HIV-1 inhibition (R5-restricted or tropism-independent) was observed in serial samples from each EU (not shown), confirming the persistence of individual restriction phenotypes.

**Table 3:** Restriction in infectivity assays.

EU code	PBMC samples tested for HIV-1 infectivity^a^	CD4 cell samples tested for HIV-1 infectivity^a^	Restricted tropism^b^
	Year (month)	Year (month)	

W276	1999 (1, 7)	1998 (1), 1999 (1, 7), 2004 (6)	Pantropic
W278	1998 (1), 2000 (4)	1998 (1), 2000 (4, 8), 2004 (6)	R5 tropic
W336	1998 (1)	1998 (1), 2001 (1, 4)	R5 tropic
B184	1998 (11)	1998 (1, 11), 2004 (1)	R5 tropic
B195	1998 (1), 1999 (1)	1998 (11), 1999 (1, 7)	R5 tropic

^a ^Cell samples tested for HIV-1 susceptibility in single-round or productive infection. ^b ^Tropism associated with HIV-1 restriction (see figure 1).

## Discussion

We have previously reported that CD4 T cells from some Vietnamese individuals who remain free of infection after several years of intravenous drug use show reduced susceptibility to HIV-1 infection [11]. Here we extended our investigations of the mechanisms underlying HIV-1 restriction in CD4 T cells and found that both entry and post-entry steps of HIV-1 replication could be affected. Interestingly, the restriction in one of these subjects also affected other lentiviruses. In addition, the restriction mechanisms persisted for several years.

The same patterns of in vitro CD4 T cell resistance to HIV-1 infection were observed after alleged interruption of at-risk behaviors, suggesting that the mechanisms of resistance in these subjects do not depend on exposure to the virus but rather might be linked to constitutive factors. It is noteworthy in this respect that heterozygous CCR5 mutations in two of the five EUs studied here (B184 and W336) were associated with low CCR5 surface expression on their primary CD4 T cells and with resistance of these cells to HIV-1 R5 infection. CCR5Δ32 heterozygosity has been associated with decreases both in CCR5 surface expression and in susceptibility to in vitro infection by R5 viruses, although to a lesser extent than CCR5Δ32 homozygosity [35-37]. Low CCR5 expression in CCR5Δ32 heterozygous cells has been attributed to several mechanisms, including sequestration of the wild-type molecule by the mutant molecule in the endoplasmic reticulum, and reduced gene dosage [37-39]. The molecular mechanisms underlying the reduced CCR5 expression in the heterozygous Vietnamese EUs' CD4 T cells are under investigation.

CD4 T cells from subjects W278 and B195 were also resistant to infection by HIV-1 R5, even though these EUs had the wild-type CCR5 molecule. HIV restriction in these subjects' cells was abrogated by anti-β-chemokine Abs. Accordingly, PCR experiments suggested that the block in CD4 T cells from subjects W278 and B195 affected very early steps of viral replication [11], likely reflecting inhibition of viral entry by β-chemokine ligands of CCR5 [40]. Partial resistance to HIV-1 R5 in cells from some CCR5-wt EUs has previously been linked to decreased CCR5 expression on the CD4 T cell surface and to increased β-chemokine secretion [12]. CCR5 expression on CD4 T cells from subjects W278 and B195 was not subnormal. However, as CCR5 expression on thawed cells (including from controls) was too low for FACS analysis, our experiments were done 10 days after PHA stimulation and we cannot therefore formally exclude the possibility that CCR5 expression was reduced on W278 and B195 CD4 T cells at the time of infection (three days after PHA stimulation) and recovered rapidly thereafter. Nevertheless, β-chemokine secretion by CD4 T cells upon mitogen activation was not higher in the two EUs than in controls, suggesting that the inhibitory mechanism differs from those previously reported. Moreover, non-stimulated CD4 T cells from these two EUs expressed normal levels of CCR5 and allowed HIV-1 entry and replication. However, in these conditions, in which endogenous secretion of β-chemokines is very low, HIV-1 infection was inhibited by exogenous β-chemokines at lower concentrations than in experiments with control cells. Thus, HIV-1 inhibition in PHA-activated CD4 T cells appears to result from enhanced sensitivity to secreted β-chemokines. In the context of wild-type CCR5, this increased sensitivity might be governed by the chemoreceptor microenvironment, which has been shown to influence both CCR5 affinity for its agonists [41] and β-chemokine-induced CCR5 internalization [42].

CD4 T cells from subject W276 exhibited a pantropic restriction phenotype independent of the virus entry pathway. Viral replication was blocked at early post-entry steps, probably through impaired reverse transcription. The restriction pattern in W276 cells (i.e. non-saturable, blockade of several lentiviruses) differed from that attributed to TRIM5α and APOBEC family proteins – restriction factors that also target early post-entry steps of viral replication [43-45]. Preliminary analyses of heterokaryons obtained by fusion of W276 CD4 T cells with the HIV-susceptible cell line A2.01 (data not shown) suggest that the restriction in EU W276 cells might be due to missing or defective cell factor(s) necessary for viral replication, rather than to antiviral molecules.

Strong CD4 T cell resistance to HIV-1 infection is a highly unusual phenomenon and it is reportedly more frequent among EUs [9,11]. These cells provide unique opportunities for identifying novel HIV-1 resistance mechanisms. For example, the CCR5Δ32 homozygous genotype was first identified in two EUs with reduced susceptibility to HIV-1 infection [15], but has since been associated with protection in Caucasians [17] and has led to the development of CCR5-targeting drugs [46]. However, CCR5Δ32 homozygosity accounts for cell resistance in only a small fraction of Caucasian EUs.

## Conclusion

Each of the in vitro resistance mechanisms described here may contribute to protection against HIV-1 infection in exposed uninfected Vietnamese individuals, possibly in conjunction with other innate or adaptive antiviral responses [47,48]. Low CCR5 expression due to CCR5 mutations in target cells may limit the infection and spread of HIV-1 R5 viruses, which are preferentially transmitted and predominate in the early phases of the human infection [49,50]. β-chemokine-mediated resistance to HIV-1 R5 infection of activated CCR5-wt CD4 T cells could limit HIV-1 transmission and spread at preferential sites of viral replication. Indeed, HIV-1 replication occurs mainly in activated CD4 T cells, which tend to be located in β-chemokine-rich environments such as lymph nodes and gut-associated lymphoid tissue [51,52]. Finally, near-complete restriction of viral replication, as found in the cells of EU subject W276, probably protects against HIV-1 transmission, as in CCR5Δ32 homozygous individuals. Identification of the mechanisms and molecules involved in such broad lentivirus restriction may lead to new viral and/or cellular targets for anti-HIV therapy.

## Materials and methods

### Study subjects

The five EUs studied here (Table 1) belonged to a population of intravenous drug users (IDU) who had been exposed to HIV-1 through needle sharing for many years [11,53]. Subjects W276, W278, and B195 correspond to subjects EU1 to EU3 and subject B184 corresponds to subject EU13 in [11]. W336 was first described in [25]. When recruited, they had been using drugs for 17 to 26 years. All continued high-risk practices for several years despite medical counseling. Four subsequently said they had stopped at-risk drug use between 2000 and 2003 (Table 1). Subject B195 was lost to follow-up in July 1999. Controls were Vietnamese (20) and European (7) healthy blood donors with a low risk of HIV-1 infection (Red Cross, Vietnam and Centre de Transfusion Sanguine Ile-de-France, Rungis, France). All the infectivity assays with EU CD4 T cells were performed in parallel with susceptible CD4 T cells from at least three randomly selected controls. All participants gave their informed consent.

### CD4 T cells

Peripheral blood mononuclear cells (PBMC) from EUs and controls were isolated from whole blood by Ficoll-Hypaque centrifugation. CD4 cells were purified from thawed PBMC by positive selection with antibody-coated immunomagnetic beads (Miltenyi Biotech, France). Activated CD4 T cells (>95% CD4+CD3+CD25+ as estimated by flow cytometry) were obtained after stimulation for three days with phytohemagglutinin (PHA, 1 μg/ml) and interleukin-2 (IL2) (Chiron, France, 100 IU/ml) and were maintained in RPMI 1640 medium containing 10% fetal calf serum, penicillin/streptomycin (100 U/ml) and IL2.

### Production of reporter viral particles and infectious challenge

Pseudotyped reporter retroviral particles were produced by transiently co-transfecting 293T cells with the proviral constructs pNL-Luc-E-R+, pSIVmac-Luc-E-R+ or pSIVagm-Luc-E-R+ [43,54] and the VSV-G, HxB2-Env, BaL-Env, JRFL-Env or YU2-Env expression vectors (7.5 μg each) using the lipofection reagent SuperFect (Qiagen, France). Supernatants were harvested 48 h after transfection, and 10^5 ^CD4 T cells were infected (m.o.i: 0.1–1.0) in triplicate in 96-well plates with a spinoculation protocol [55] (1 hour of centrifugation at room temperature at 1500 *g*, followed by 1 hour at 37°C). After challenge, cells were extensively washed and then cultured.

### Quantification of luciferase activity in cell lysates

Three days after challenge the cells were harvested and lysed with 100 μl of luciferase lysis buffer (Promega, France). Luciferase activity was quantified in 10 μl of each lysate with the Promega Luciferase Assay System in a Veritas microplate luminometer (Turner BioSystems, CA, USA).

### CCR5 genotypic characterization

DNA was extracted from PBMC with the DNeasy Tissue Kit (Qiagen, Courtaboeuf, France). The full-length coding region (exon 4) of the CCR5 gene was amplified with primers and in conditions described elsewhere [24].

PCR products were purified with the ExoSAP-IT^® ^enzyme for PCR Product Clean-Up (Pharmacia-Amersham, USA) and were directly sequenced with the BigDye Terminator cycle sequencing kit (ver.3.1; Applera, France). Sequences were determined with an automatic sequencer (ABI-Prism 3100, Applied Biosystem, USA) and analyzed with SeqScape software version 2.5 (Applied Biosystem, USA).

### Flow cytometry of CCR5 expression

Ten days after PHA activation, CD4 T cells were incubated for 30 minutes at room temperature with CCR5-FITC (clone 2D7) (BD Bioscience, France) and analyzed on a Cytomics FC500 flow cytometer (Beckman Coulter, Paris, France).

### CCR5-mediated actin polymerisation

Actin polymerization in CD4 T cells was measured as described elsewhere [30]. Briefly, ten days after PHA stimulation, cells (1 × 10^7 ^cells/mL) were incubated in RPMI medium containing 20 mM HEPES in the presence or absence of inhibitor. RANTES (30 nM) was then added to the cell suspension. At each indicated time point (15 s to 2 min), a 50-μL aliquot of cell suspension was mixed with 200 μL of labeling buffer consisting of 10^-7 ^M FITC-phalloidin (Sigma), 0.125 mg/mL L-α-lysophosphatidylcholine palmitoyl (Sigma) and 4.5% PFA in PBS. The kinetics of actin polymerization was monitored by means of flow cytometry. Results are expressed as follows: [MFI after addition of ligand/MFI before addition of ligand] × 100]. MFI values before ligand addition were arbitrarily set at 100%. Owing to the large number of cells required, CD4 T cells were amplified on irradiated heterologous feeder PBMC for two weeks prior to testing. The pattern of HIV-1 restriction in amplified cells was similar to that found in the primary CD4 T cells (not shown). TAK-779 was obtained through the NIH AIDS Research and Reference Reagent Program, Division of AIDS, NIAID, NIH.

### Quantification of secreted β-chemokines

Levels of β-chemokines, RANTES, MIP-1α and MIP-1β in the supernatants of CD4 T cells were measured after 72 h of culture with or without PHA stimulation, by using commercial ELISA kits (Quantikine, R&D systems, France).

### Real-time PCR quantification of HIV-1 replication intermediates

Three days after PHA stimulation, CD4 T cells were challenged with DNase (Invitrogen, France)-pretreated viruses (1 h at room temperature). At the times indicated, 5 × 10^5 ^cells were washed in PBS and lysed, then total DNA was extracted with the DNeasy Tissue Kit (Qiagen, France). Early HIV-1 reverse transcription products were quantified with an ABI PRISM 7000 instrument (Applied Biosystems, France) using specific primers and probe as previously described [56]. One hundred nanograms of template DNA was used per reaction, and the albumin gene was used as a housekeeping gene to normalize sample input. 8E5 cells containing one integrated copy of HIV-1 per cell [57] were used to construct standard curves.

## Competing interests

The author(s) declare that they have no competing interests.

## Authors' contributions

ASC, LT, FBS and GP conceived the study and contributed to its experimental design and coordination. DSA participated in the design of the study. ASC, PV, LC and WC performed the experiments. ASC, LC, WC, DSA and GP participated in the data analysis. ASC and GP drafted the manuscript. All the authors critically reviewed and approved the final manuscript.
